# Study on the Critical Value of Residual Gas Content Based on the Difference of Adsorption Structure between Soft and Hard Coal

**DOI:** 10.1021/acsomega.1c00533

**Published:** 2021-06-23

**Authors:** Minmin Li, Weimin Liang, Haixiao Lin, Gaowei Yue

**Affiliations:** School of Civil Engineering, Henan Polytechnic University, Jiaozuo 454000, Henan, China

## Abstract

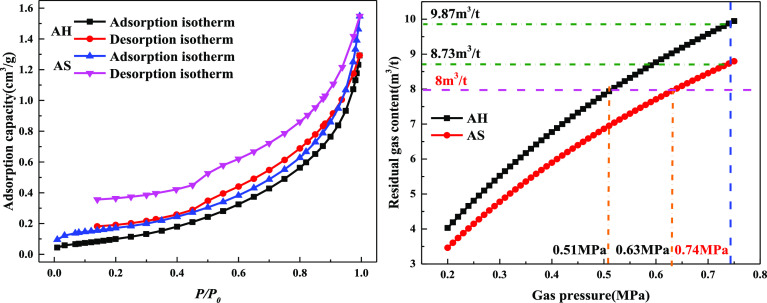

In view of the difference of the adsorption structure between soft and hard coal, there is a big difference in the critical value of the inspection index for the regional outburst risk caused by the gas content. For the coal seams with soft and hard coal stratification, the model of gas content in the equilibrium state was established first, and the microscopic parameters of different rank coals were determined by the low-temperature liquid nitrogen adsorption test and mercury intrusion test. Then, the adsorption capacity of coal samples was determined by the adsorption test. Finally, the residual gas content of the coal seam in the equilibrium state was calculated based on the adsorbed gas content, and the critical value of prediction indexes of regional outburst based on the residual gas content was studied. The results show that for the same metamorphic degree, the specific surface area of soft coal is larger than that of hard coal. However, under the same gas pressure, the residual gas content of hard coal of anthracite and lean coal is greater than that of soft coal with the same metamorphic degree, while that of meager-lean coal and gas-fat coal is opposite. It is suggested to adopt the small value (rounded) of the measured gas content of soft and hard coal at 0.74 MPa as the critical value of the residual gas content in the regional effect test from the economic perspective. It is of great significance to determine the critical standard of the residual gas content in the regional effect test according to local conditions for reducing the cost of outburst prevention work.

## 1 Introduction

In China, the *Regulations on Prevention and Control of Coal and Gas Outburst* stipulates that the critical values of gas pressure and gas content should be 0.74 MPa and 8 m^3^/t, respectively, without experimental investigation.^1^ However, when the soft and hard coals coexist in the coal seam, the prediction of coal and gas outburst is carried out according to these critical values, and it is found that the prediction index is not sensitive and the critical value is unreliable sometimes.^2−5^ Many coal seams in mines often contain soft and hard coal stratification. There are significant differences in mechanical characteristics and permeability between soft and hard coal.^6−9^ The mechanical strength of soft coal stratification is low, which is a prominent breakthrough point.^10−12^ Therefore, based on the difference of adsorption structure between soft and hard coal, it is of great significance to study the critical value of the inspection index for the regional outburst risk caused by the residual gas content when soft and hard coals coexist.

There are obvious differences in the pore structure between soft and hard coal.^13−16^ Yang et al.^17^ discussed the difference between soft and hard coal from the aspects of pore size distribution, pore structure, specific surface area, and permeability and pointed out that in hard coal, macropores with a pore size greater than 50,000 nm account for about 50%, while micropores with a pore size less than 100 nm account for about 41.23% in soft coal. The surface area of soft coal is 1.6 times that of hard coal. At the same time, the gas adsorption capacity of soft and hard coal is also different.^18−20^ Yuan^21^ studied in detail the characteristics of gas diffusion and migration between soft and hard coal from three research perspectives of macrostructure, mesostructure, and microstructure and explored the root cause of the difference in gas diffusion and migration. For the macroscopic structure, the particles of soft coal are flaky and nail shaped. At the same time, it is observed that the particles of hard coal form complete lumps. In addition, for the mesoscopic structure, it is found that the proportion of soft coal with particle size less than 6 mm is much higher than that of hard coal. For the microscopic characteristics, pores and cracks are observed on the surface of soft coal, and the BJH specific surface area of soft coal is more than twice that of hard coal, which means that the gas diffusion and migration conditions of soft coal are better than those of hard coal. Sun et al.^2^ introduced the adsorption and desorption rule of soft and hard coal in the outburst-prone coal seam and the prediction index of their sensitivity to outburst. It is pointed out that the initial desorption rate of soft coal is faster than that of hard coal, and the outburst prediction index K1 is more reliable than Δ*H*2. Liu et al.^22^ discussed the difference of molecular structure between deformed soft coal and hard coal on methane adsorption. The results showed that the molecular structure of the deformed soft coal is significantly different, that is, compared with that of hard coal, the interlayer spacing *d*_002_ of the deformed soft coal is smaller, and the lateral dimension *La*, stacking height *Lc*, and crystal nucleus size *La*/*Lc* are larger. The adsorption capacity of the deformed soft coal with the same rank coal is larger. Liu et al.^23^ used Fourier transform infrared spectroscopy (FTIR) and X-ray photoelectron spectroscopy (XPS) to study soft and hard coal with metamorphism from bituminous coal to anthracite. The results showed that run coal has a larger aromatic ring and a higher maturity than the corresponding hard coal, showing the evolution characteristics of early maturity. Mastalerz et al.^24^ considered that the average limit adsorption value of soft coal is 21.26%–74% higher than that of the primary structure coal. This leads to the phenomenon that the coal seams with soft and hard coal stratification cannot eliminate the outburst at the same time when the effect of outburst prevention measures is tested. Moreover, the critical values of inspection indexes of the regional outburst prevention with different metamorphic degrees are greatly different, and the inspection indexes are copied, resulting in the phenomenon that the gas does not burst when the index is high, and the gas bursts when the index is low. Therefore, it is of great practical significance to study the critical value of the inspection index in the regional effect test in line with the actual mine.

In this paper, the coal samples with different metamorphic degrees (anthracite, lean coal, meager-lean coal, and gas-fat coal) and coal samples with different failure types (hard coal and soft coal) are selected to measure the basic parameters related to the outburst of soft and hard coal. The difference in pore structure and adsorption law of soft and hard coal is discussed. At the same time, the relationship model of related parameters is established, and the critical value of the inspection index for the regional outburst risk caused by the residual gas content is studied.

## 2 Results and Discussion

### 2.1 Low-Temperature Liquid Nitrogen Adsorption/Desorption Test

**Figure 1:**
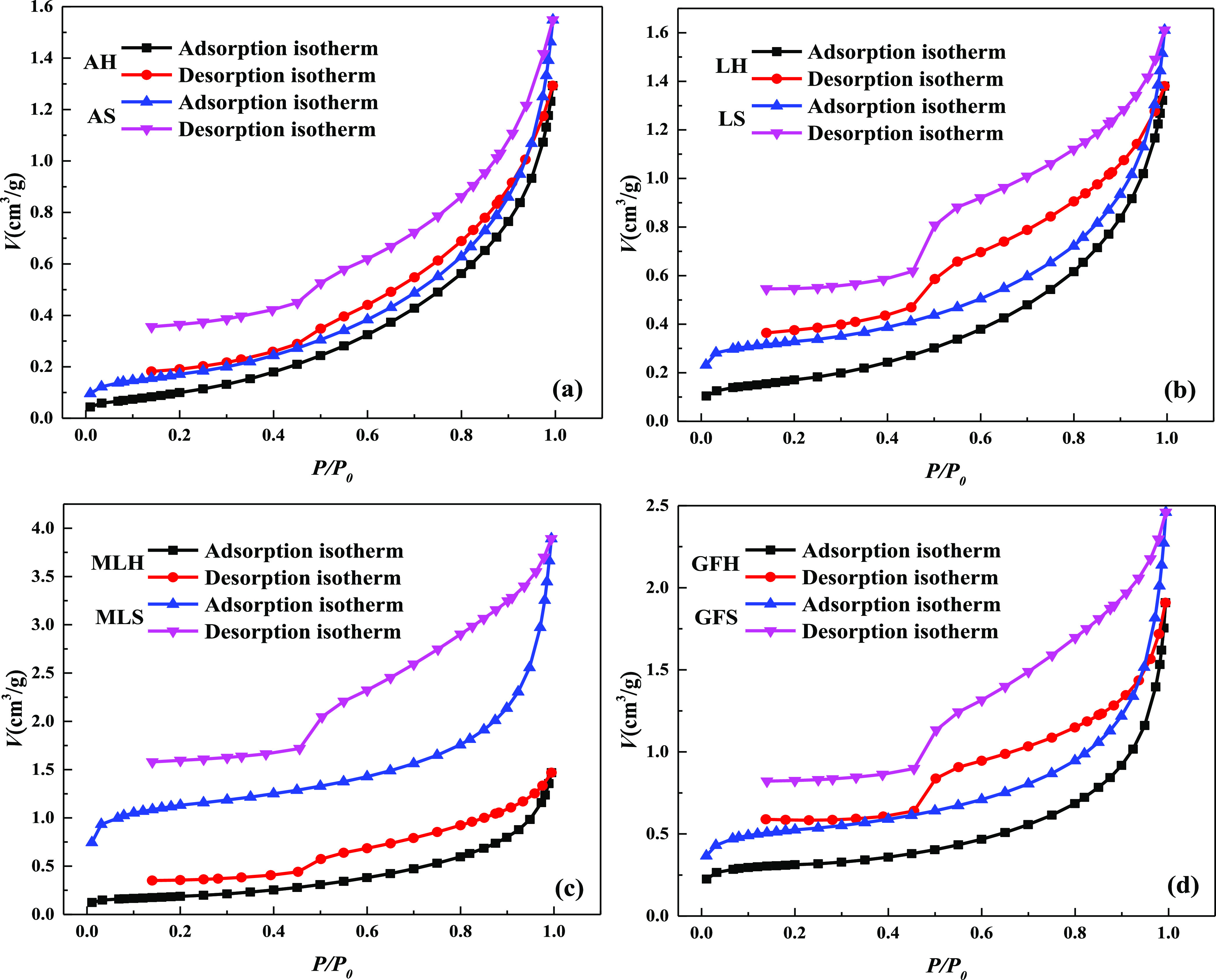
Adsorption/desorption curves of different rank coals: (a) anthracite, (b) lean coal, (c) meager-lean coal, and (d) gas-fat coal.

The pore range measured by the low-temperature liquid nitrogen adsorption/desorption test is small, which mainly measures the specific surface area of micropores. The adsorption/desorption curves of different rank coals are shown in Figure 1. The adsorption curve rises slowly and steadily, and an obvious hysteresis loop is found in the desorption curve, which is caused by the obvious difference of the pore throat between pores, and the development of pores is mainly micropores. When the relative pressure is 0.5 MPa, there is an obvious inflection point, which reflects that the pore type is “ink bottle” with open and good connectivity. This type of pore first condenses in the bottle neck, and the gas–liquid surface is cylindrical. With the increase of adsorption capacity, the bottle is finally filled with condensed liquid. When the relative pressure decreases, the condensed liquid in the bottle neck is sealed in the bottle, so the evaporation effect cannot occur, resulting in a hysteresis loop. Then, the thin neck begins to evaporate as the relative pressure increases. At this time, the gas–liquid surface is hemispherical. After the liquid in the bottle neck evaporates, the condensed liquid in the bottle evaporates rapidly, so there is a significant inflection point on the desorption curve.

**Figure 2:**
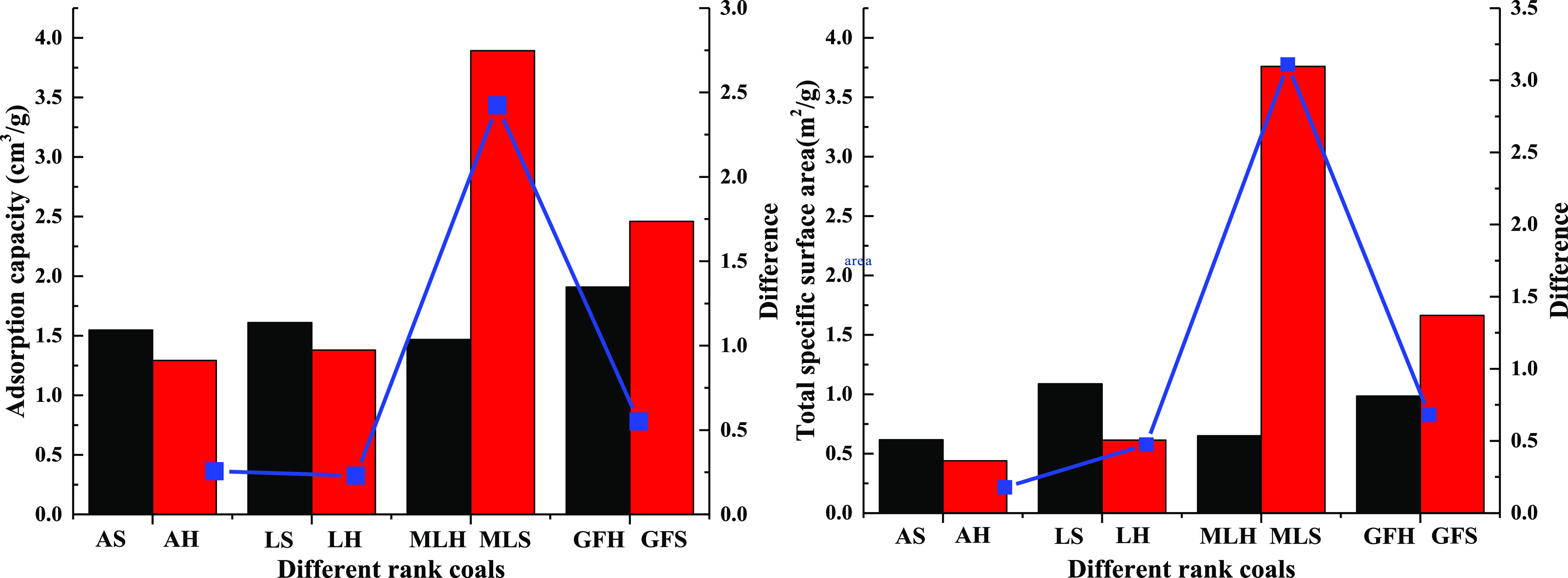
Difference of adsorption capacity and specific surface area of different rank coals.

According to the adsorption isotherm, the specific surface area of the coal sample is obtained, as shown in Table 1. The difference of the adsorption capacity and specific surface area of different rank coals is shown in Figure 2. The adsorption capacity and total specific surface area of soft and hard coal with different rank coals are quite different. The difference of adsorption capacity and specific surface area of soft and hard coal of meager-lean coal is the largest. The difference of adsorption capacity of soft and hard coal of meager-lean coal is 9.47 times, 10.53 times, and 4.4 times that of anthracite, lean coal, and gas-fat coal, respectively. The difference of the specific surface area of soft and hard coal of meager-lean coal is 17.47 times, 6.56 times, and 4.58 times that of anthracite, lean coal, and gas-fat coal, respectively. In the same coal ranks, the adsorption capacity and total specific surface area of soft coal are larger than that of hard coal. The difference of adsorption capacity and specific surface area of different rank coals has a similar change law, which increases first and then decreases with the increase of the rank coal.

**Table 1:** Test Results of Coal Samples

coal sample	adsorption capacity (cm^3^/g)	total specific surface area (m^2^/g)	pore volume (cm^3^/g)	mercury removal efficiency
AH	1.2924	0.4396	0.0503	0.1638
AS	1.5482	0.6175	0.0536	0.1742
LH	1.3801	0.6141	0.0430	0.1625
LS	1.6103	1.0883	0.0455	0.1671
MLH	1.4688	0.6503	0.0303	0.1824
MLS	3.8919	3.7591	0.0344	0.1543
GFH	1.9089	0.9848	0.0292	0.1959
GFS	2.4599	1.6643	0.0294	0.1596

### 2.2 Mercury Intrusion Test

**Figure 3:**
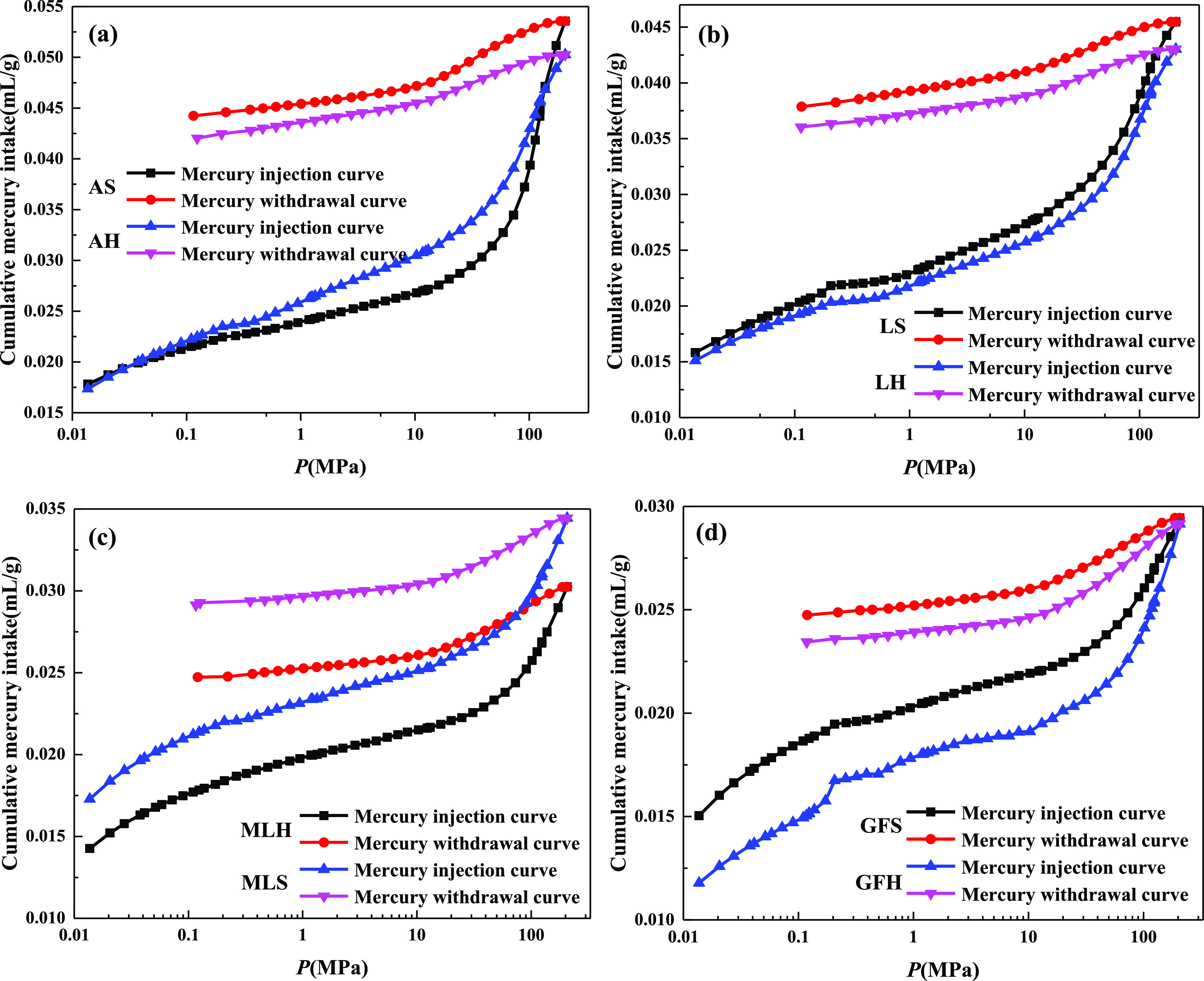
Mercury injection/withdrawal curves of different rank coals: (a) anthracite, (b) lean coal, (c) meager-lean coal, and (d) gas-fat coal.

The pore range measured by the mercury intrusion test has a wide range of pores, which is suitable for the measurement of medium to large pores. The mercury injection/withdrawal curves of different rank coals are shown in Figure 3. The mercury injection/withdrawal curve can reflect the pore structure and connectivity of coal. The effective pore structure in coal mainly includes two basic types, open pore and semiclosed pore. The higher the proportion of open pores, the better the connectivity and openness of the coal. The higher the proportion of semiclosed pores, the worse the connectivity and openness of the coal. In terms of mercury intake, anthracite has the most mercury intake and gas-fat coal has the least. Because the mercury intake reflects the pore volume of the reservoir, it is obvious that the pore space of anthracite is more developed than that of other rank coals. The injection and withdrawal mercury curves of coal samples are not coincident, and there is a certain hysteresis phenomenon, which can effectively reflect the basic shape and connectivity of coal samples. The hysteresis loop of the mercury injection curve of anthracite is the widest, and the difference of volume between the injection and withdrawal of mercury is large, which indicates that the pore shape is mainly open pores, with more “ink bottle” pores and strong connectivity, which is very conducive to the desorption, diffusion, and seepage of coalbed methane.

**Figure 4:**
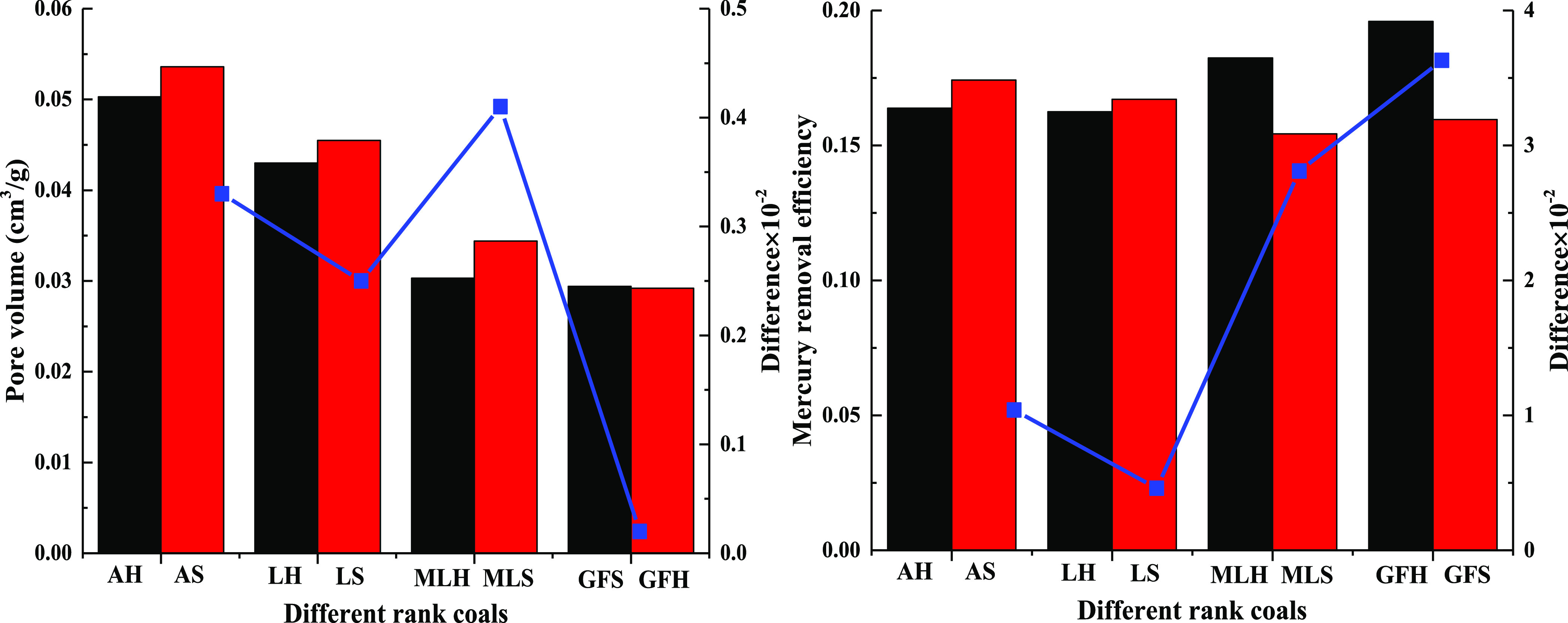
Difference of pore volume and mercury removal efficiency of different rank coals.

The pore volume and mercury removal efficiency of different rank coals are shown in Table 1 and Figure 4. The higher the connectivity of pores, the better the mercury removal efficiency. The difference of pore volume of soft and hard coal of meager-lean coal is the largest, and the difference of mercury removal efficiency of soft and hard coal of gas-fat coal is the largest. The difference of pore volume of soft and hard coal of meager-lean coal is 4.61 times, 10.13 times, and 72 times that of anthracite, lean coal, and gas-fat coal, respectively. The difference of mercury removal efficiency of soft and hard coal of gas-fat coal is 3.49 times, 7.89 times, and 1.29 times that of anthracite, lean coal, and meager-lean coal, respectively.

### 2.3 Adsorption Characteristics of Soft and Hard Coal

**Figure 5:**
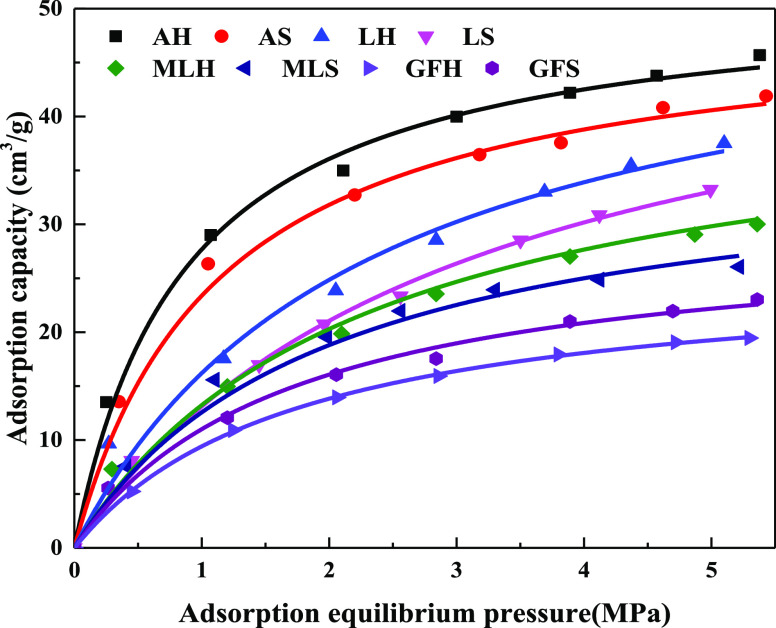
Absorption isotherms of coal samples.

The adsorption curves of soft and hard coal with different metamorphic degrees are drawn through the experimental data of isothermal adsorption, as shown in Figure 5. There is little difference in the adsorption capacity of soft and hard coal with the same metamorphic degrees. For example, for anthracite, the adsorption capacity of hard coal is 0.89–1 times that of soft coal; for lean coal, the adsorption capacity of hard coal is 0.82–0.97 times that of soft coal; for meager-lean coal, the adsorption capacity of hard coal is 0.86–1.04 times that of soft coal; and for gas-fat coal, the adsorption capacity of hard coal is 1.06–1.18 times that of soft coal. For coal samples with different metamorphic degrees, the differences of adsorption capacity of soft and hard coal are not the same. When the gas pressure is high, the gas adsorption capacity is anthracite, lean coal, meager-lean coal, and gas-fat coal in order. The Langmuir adsorption curve is used to fit the adsorption capacity. The adsorption constants of different rank coals are shown in Table 2. It can be seen from Table 1 and Figure 5 that the specific surface area of soft coal differs several times from that of hard coal with the same metamorphic degree, while the adsorption capacity of soft coal slightly differs from that of hard coal. Meanwhile, the adsorption capacity of hard coal with a much smaller specific surface area and total pore volume is larger than that of soft coal with a larger specific surface area and total pore volume, such as anthracite, lean coal, and meager-lean coal.

**Table 2:** Adsorption Constant of Coal Samples

	adsorption constant		
coal sample	*a* (cm^3^/g)	*b* (MPa^–1^)	*R*^2^
AH	51.20	1.22	0.995
AS	47.76	1.10	0.996
LH	53.30	0.44	0.981
LS	53.23	0.33	0.997
MLH	40.76	0.50	0.992
MLS	33.42	0.67	0.999
GFH	26.06	0.57	0.999
GFS	29.71	0.59	0.991

### 2.4 Critical Value of the Residual Gas Content in the Coal Seam

Affected by gas drainage, the distribution of gas pressure in coal seams is uneven, that is, there are gas pressure gradients of soft coal and hard coal stratification in the same coal seam. However, in a period of time after gas drainage, the gas pressure of soft and hard coal will tend to be the same. Due to the difference of pore structure and gas adsorption capacity between soft and hard coal, the residual gas content is bound to be different.

**Figure 6:**
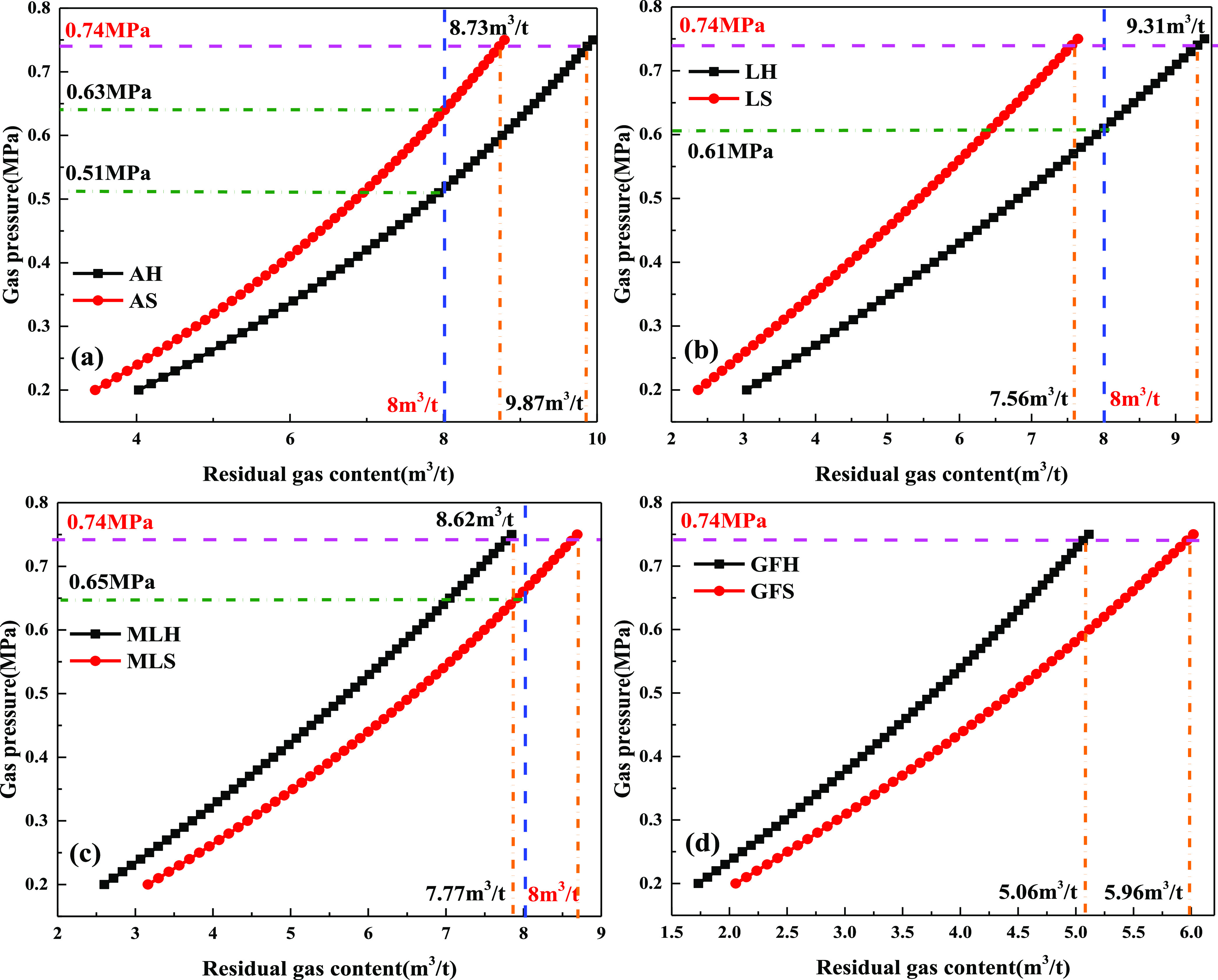
Variation law of residual gas content and gas pressure: (a) anthracite, (b) lean coal, (c) meager-lean coal, and (d) gas-fat coal.

The critical value of gas pressure in the *Regulations on Prevention of Coal and Gas Outburst* is 0.74 MPa. When the coal seam temperature is 30 °C, the residual gas content in coal seams with different metamorphic degrees can be calculated according to Formula 7, as shown in Figure 6. As the residual gas pressure increases, the gas pressure also increases. Under the same gas pressure, the residual gas content of hard coal of anthracite and lean coal is greater than that of soft coal, while that of meager-lean coal and gas-fat coal is opposite. The main reason may be that under the same adsorption equilibrium pressure, the thickness of the methane adsorption layer changes negatively exponentially with the increase of pore size, and the smaller the gas adsorption equilibrium pressure is, the thinner the adsorption layer is. Methane has more adsorption layers in small pores but less in large pores. The adsorption capacity of soft and hard coal depends on the number of adsorption layers in different pore sizes but has no positive correlation between the specific surface area and total pore volume.^14^ At the same time, it can be seen from eq 7 that the gas content of the coal is mainly determined by the gas adsorption content. Therefore, the variation law of residual gas content in coal seam with different metamorphic degrees is different with gas pressure.

When the gas pressure in the coal seam is 0.74 MPa, the residual gas content is different. The gas content of anthracite with high metamorphism reaches 9.87 m^3^/t, followed by lean coal, which reaches 9.31 m^3^/t. If the gas content of 8 m^3^/t is taken as the critical value of the inspection index of the regional outburst prevention, the residual gas content in soft and hard coal with different metamorphic degrees should reach the standard below 8 m^3^/t. Under this standard, the minimum gas pressure is 0.51 MPa when anthracite is extracted, while that of lean coal is 0.61 MPa.

As a result, there is a very tangled problem. The reasons are as follows: (1) when 0.74 MPa is used as the critical value of gas pressure in the soft and hard coal seam, the content of residual gas in the coal seam is often more than 8 m^3^/t. (2) When 8 m^3^/t is used as the critical value of residual gas in the soft and hard coal seam, the gas pressure in the coal seam is often much lower than 0.74 MPa. If we do not fully consider the actual conditions according to local conditions, only blindly imitate or copy the previous methods and experience, it will seriously affect the safety and efficient mining of the coal mine and even cause the imbalance of mining.

In order to reduce the unnecessary engineering investment in the early stage and the blindness in the implementation process of predrainage in the coal seam, for soft and hard coal stratification with different metamorphic degrees, it is suggested to adopt the small value (rounded) of the gas content measured in soft coal and hard coal at 0.74 MPa as the critical value of residual gas content in the coal seam. Therefore, the residual gas content should be less than 8 m^3^/t for anthracite, less than 7 m^3^/t for lean coal and meager-lean coal, and less than 5 m^3^/t for gas-fat coal. This will provide guidance for the inspection index of the residual gas content in outburst elimination in the coal seam with soft stratification.

### 2.5 Field Application

**Figure 7:**
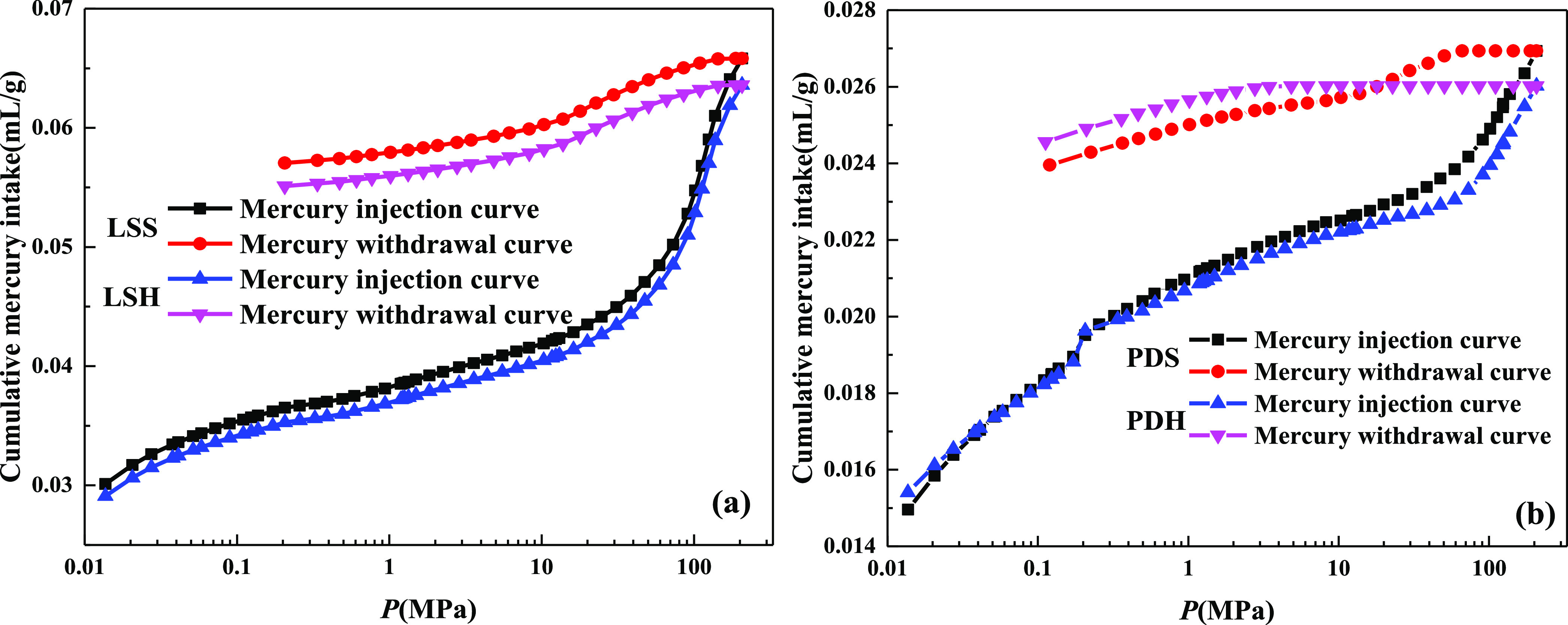
Mercury injection/withdrawal curve of soft and hard coal: (a) Longshan mine and (b) Pingmei no. 1 mine.

**Figure 8:**
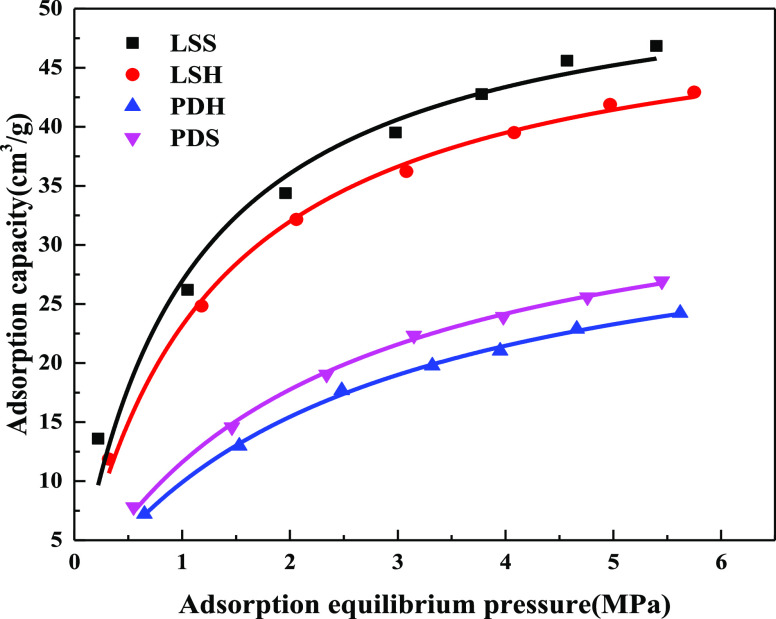
Adsorption isotherms of soft and hard coal.

Two kinds of metamorphic coals were selected for field verification. Longshan mine is located in the middle of the Anhe coalfield in Henan Province. It mainly mines the second-1 coal seam, with an average thickness of 4.5 m, an average volatile content of 6.5%, and a maximum reflectivity of 2.89%. The type of coal is highly metamorphic anthracite. Pingmei no. 1 mine is located in the middle of the Pingdingshan mining area. Ding-6 coal seam is one of its main coal seams. The thickness of the coal seam is 0.25–3.79 m, with an average thickness of 2.2 m. The type of coal is mainly gas-fat coal, with a volatile content of 34.29%. Samples were taken from the coal seam with soft and hard stratification, and industrial analysis and parameter test of coal samples were carried out, as shown in Table 3, and among them, LSS and LSH represent the soft and hard coal of the Longshan mine; PDS and PDH represent the soft and hard coal of the Pingmei no. 1 mine. Mercury injection and withdrawn curves of soft and hard coal in the Longshan mine and Pingmei no. 1 mine are shown in Figure 7. The isothermal adsorption tests were conducted at 30 °C, as shown in Figure 8. According to the Langmuir fitting data, the a values of soft and hard coal in the Longshan mine are 51.62 and 54.43 cm^3^/g, respectively, and the hard coal is 5.16% higher than the soft coal. The *b* values of soft and hard coal are 0.81 and 0.98 MPa^–1^, respectively, and the hard coal is 17.35% higher than the soft coal. In Pingmei no. 1 mine, the a values of soft and hard coal are 37.86 and 35.15 cm^3^/g, respectively, and the soft coal is 7.16% higher than the hard coal; the b values of soft and hard coal are 0.44 and 0.39 MPa^–1^, respectively, and the soft coal is 11.36% higher than the hard coal.

**Table 3:** Basic Parameters of Soft and Hard Coal

	industrial analysis				
coal sample	*M*_ad_ (%)	*A*_ad_ (%)	*V*_daf_ (%)	pore volume (cm^3^/g)	total specific surface area (m^2^/g)
LSH	3.74	11.75	11.05	0.0636	0.3001
LSS	3.62	10.81	9.94	0.0658	0.5715
PDH	1.17	11.81	33.03	0.0260	0.5202
PDS	1.21	12.17	30.67	0.0269	0.5553

**Figure 9:**
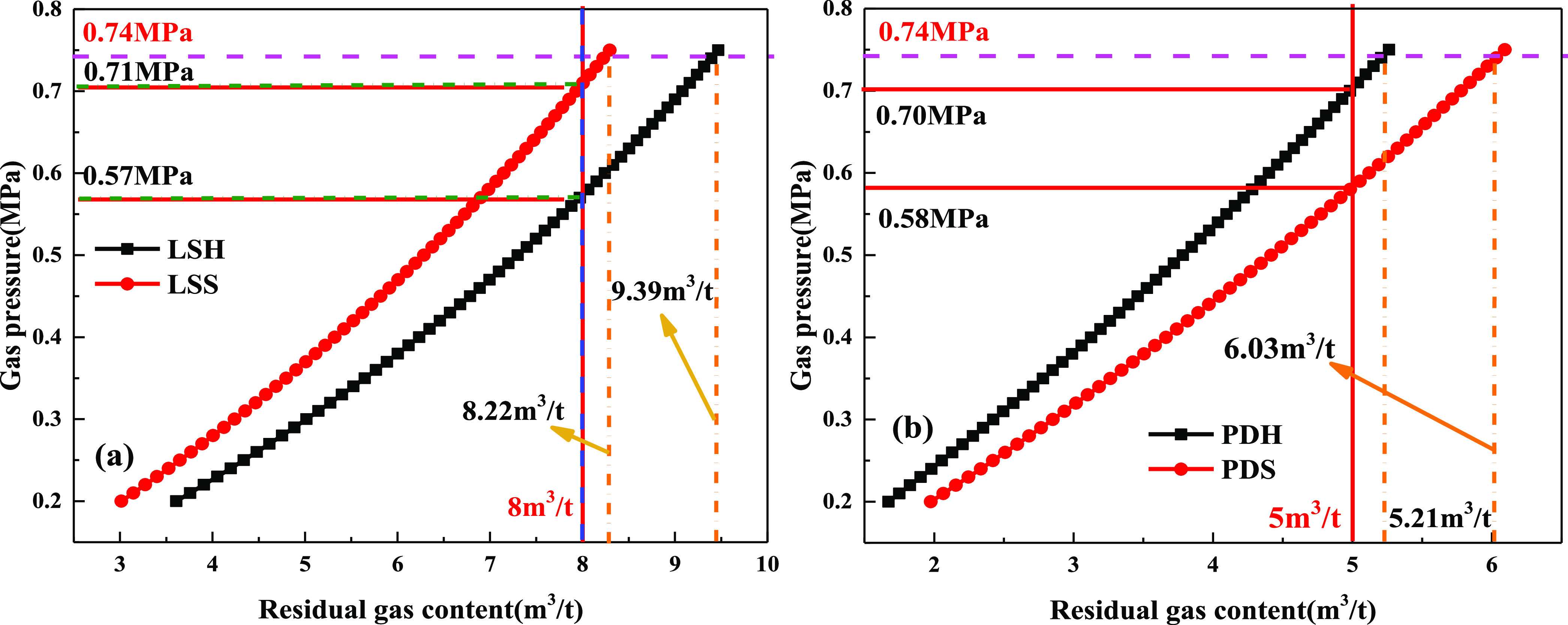
Variation law of the residual gas content and gas pressure: (a) Longshan mine and (b) Pingmei no. 1 mine.

When the critical value of gas pressure is 0.74 MPa, the residual gas content of the soft and hard coal in the Longshan mine can be calculated as 8.22 and 9.39 m^3^/t, respectively, and the residual gas content of the soft and hard coal in the Pingmei no.1 mine can be calculated as 6.03 and 5.21 m^3^/t, respectively, as shown in Figure 9. For the Longshan mine and Pingmei no. 1 mine, the abovementioned suggested method is used to take the small value of the residual gas content at 0.74 MPa and approximately, namely, 8 and 5 m^3^/t as the inspection index of residual gas content in the predrainage and outburst elimination. At this time, in the Longshan mine, when 8 m^3^/t is used as the inspection index of residual gas content, the corresponding gas pressures of soft and hard coal are 0.71 and 0.57 MPa, respectively. In Pingmei no. 1 coal mine, when 5 m^3^/t is used as the inspection index of residual gas content, the corresponding gas pressures of soft and hard coal are 0.58 and 0.70 MPa, respectively.

After a period of drainage time, soft coal stratification and hard coal bottom drilling were carried out at 41.8 m away from the middle line of 11011 upper auxiliary roadway in the Longshan mine. The measured gas contents of soft and hard coal are 7.89 and 9.22 m^3^/t, respectively. According to Formula 6, the gas pressures are 0.69 and 0.72 MPa, respectively, which meet the inspection index of regional outburst prevention measures in the *Regulations on Prevention and Control of Coal and Gas Outburst*. In Pingmei no. 1 coal mine, the soft coal stratification and hard coal bottom drilling were carried out at a distance of 30 m from the panel opening of 32,060 machine lane. The measured gas contents of soft and hard coal are 5.96 and 5.18 m^3^/t, respectively. According to Formula 6, the gas pressures are 0.73 and 0.61 MPa, respectively, which meet the inspection index of the regional outburst prevention measures in *Regulations on Prevention and Control of Coal and Gas Outburst*. Therefore, it is feasible to determine the critical value of the inspection index of regional outburst prevention measures by the above mentioned method in the coal seam with soft and hard stratification.

When inspecting the effect of regional outburst prevention in coal seams with soft and hard coal, the engineering phenomenon that “Hard coal stratification eliminates outburst, while soft coal stratification does not eliminate outburst” often appears in the same detection unit. In this paper, from the perspective of the difference of adsorption structure between soft and hard coal, the critical value of the regional outburst prevention measure based on the residual gas content in coal seams with different metamorphic degrees is studied. From the perspective of safety, it is best to meet the critical value of gas pressure and gas content which are lower than 0.74 MPa and 8 m^3^/t, respectively. From the perspective of economy, when the critical value of the residual gas content is 8 m^3^/t, it is often found that the gas pressure corresponding to soft and hard coal is low, and the influence factors such as extraction technology and extraction capacity as well as the porosity, permeability coefficient, and adsorption capacity of soft and hard coal are often difficult to achieve. Therefore, it is suggested that the small value (rounded) of the measured gas content of soft and hard coal at 0.74 MPa should be taken as the critical value of residual gas content in the coal seam. Therefore, it is of great significance to reduce the outburst prevention cost to determine the critical value of the inspection index of the regional outburst prevention measures in accordance with the actual mine conditions by fully considering the actual underground conditions.

## 3 Conclusions

In this paper, the structural parameters of coal samples with different rank coals are measured by the low-temperature liquid nitrogen adsorption test, mercury intrusion test, and adsorption test, respectively, and a method to determine the critical value of the inspection index of the regional outburst prevention measures in coal seams with soft and hard stratification is proposed. The results show the following:

(1) For the same metamorphic coal, the total specific surface area of soft and hard coal is several times different, and the pore specific surface area of soft coal is larger than that of hard coal.
(2) Under the same gas pressure, the gas adsorption capacity of soft and hard coal with different rank coals is significantly different. For anthracite and lean coal, the residual gas content of hard coal is greater than that of soft coal with the same metamorphic coal, while that of meager-lean coal and gas-fat coal is opposite.
(3) In view of the phenomenon that the adsorption capacity of hard coal with a smaller specific surface area and total pore volume is larger than that of soft coal with a larger specific surface area and total pore volume, there are the coal seams with soft and hard stratification, and it is suggested to adopt the small value (rounded) of the measured gas content of soft and hard coal at 0.74 MPa as the critical value.
(4) For the Longshan mine and Pingmei no. 1 mine, the proposed method is adopted to take gas contents of 8 and 5 m^3^/t, respectively, as the critical value of the inspection index of the residual gas content during pre-extraction and elimination evaluation. After draining for a period of time, the test results of the gas content have met the inspection index of the regional outburst prevention measures.

## 4 Experimental Section

### 4.1 Materials

In this experiment, coal samples with different metamorphic degrees were selected. Anthracite was taken from no. 2 coal seam of Jiulishan coal mine in Jiaozuo, Henan Province. The lean coal was selected from no. 3 coal seam of Xinyuan coal mine in Yangquan, Shanxi Province. The meager-lean coal was selected from no. 2 coal seam of Hebi coal mine, Henan Province. The gas-fat coal was selected from no. 5 coal seam of Panbei coal mine in Huainan, Anhui Province. Meanwhile, the corresponding hard and soft coals were collected according to different failure types. In the following, the hard and soft coals of anthracite are referred to as AH and AS, respectively. The hard and soft coals of the lean coal are referred to as LH and LS, respectively. The hard and soft coals of the meager-lean coal are referred to as MLH and MLS, respectively. The hard and soft coals of the gas-fat coal are referred to as GFH and GFS, respectively. Hard coals were taken by the grooving method, and then, the standard raw coal samples with a diameter of 50 mm and a height of 100 mm were prepared in the laboratory for mechanical analysis. Due to the influence of a strong geological structure, soft coal has very low strength and can be turned into a powder by hand twist, which cannot be processed into a raw coal sample. If the briquette is prepared for mechanical analysis, the mechanical parameters obtained are of little significance. Therefore, only the mechanical parameters of hard coals were provided in this paper, fresh coal samples were sealed and sent to the laboratory, and the consistent coefficient (*f*), moisture (*M*_ad_), ash content (*A*_ad_), volatile matter (*V*_daf_), uniaxial compression (σ_d_), elastic modulus (*E*), Poisson’s ratio (μ), and tensile strength (σ_t_) of the samples were determined, as shown in Table 4.

**Table 4:** Basic Parameters of Coal Samples

	industrial analysis			mechanical analysis				
coal sample	*M*_ad_ (%)	*A*_ad_ (%)	*V*_daf_ (%)	σ_d_/MPa	*E*/MPa	μ	σ_t_/MPa	*f*
AH	3.65	12.84	8.11	16.49	5.3	0.369	1.859	1.16
AS	3.47	15.24	8.69					0.38
LH	1.15	5.56	11.25	17.01	3.24	0.25	2.482	0.85
LS	1.04	6.87	12.79					0.15
MLH	1.01	8.73	15.74	15.24	4.25	0.358	2.014	0.40
MLS	0.88	10.29	15.36					0.10
GFH	1.17	11.81	33.13	19.58	2.91	0.269	2.256	0.79
GFS	1.21	12.17	30.67					0.22

### 4.2 Low-Temperature Liquid Nitrogen Adsorption/Desorption Test

The accurate and universal method for determining the specific surface area of solid is to measure the adsorption capacity of nitrogen at liquid nitrogen temperature. In this experiment, the TriStar3020 automatic fast specific surface and pore analyzer was used, and the measuring range of the pore size is 0.35–500 nm. The Brunauer–Emmett–Teller equation is the most widely used method to calculate the specific surface area, which can be obtained from the adsorption isotherm, as follows
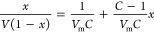
1where *V* is the adsorption capacity corresponding to the relative pressure *x*; *V*_m_ is the saturated adsorption capacity of the single molecular layer; *C* is a physical quantity related to the difference between the adsorption heat of the first layer and the condensation heat; and when the adsorbate, adsorbent, and adsorption equilibrium temperature are selected, *C* is a constant.

In the low-temperature liquid nitrogen adsorption test, all the coal samples used are granular, the particle size is 60–80 mesh, and the mass is about 3 g. The experimental process is as follows: (1) sample weighing. (2) sample degassing. The coal sample is put into the degassing station for the vacuum degassing treatment. (3) The degassed sample tube is put on the isothermal jacket and installed to the analysis port of the analysis station. (4) The dewar bottle is filled with enough liquid nitrogen, and the relative pressure is controlled in the range of 0.050–0.995. (5) The security shield is closed and then one should wait for analysis.

### 4.3 Mercury Intrusion Test

The mercury intrusion method is a standard method for measuring the distribution of macropores and mesopores. In this experiment, the AutoPore IV 9500 V1.09 automatic mercury porosimeter was used. The measurement range of the aperture is 6 nm–370 μm. The amount of mercury intrusion corresponds to the internal pore volume of the solid. There is a functional relationship between the pressure applied to mercury and the internal pore size of the solid, which conforms to the Washbum equation, namely,
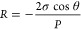
2where *R* is the pore radius, m; *P* is the injection pressure of mercury, MPa; θ is the contact angle between mercury and the tested sample, which is usually set at 140°; and σ is the surface tension of mercury, N/m, which is often taken as 0.48 N/m in the experiment.

In the mercury intrusion test, all the coal samples used are granular, the particle size is 40–60 mesh, and the mass is about 2 g. The experimental process is as follows: (1) the samples were pretreated in advance. (2) Loading the sample. With the dilatometer capillary facing down, the dilatometer is held by hand and the sample is slowly poured into the head of the dilatometer. (3) Sealing dilatometer. The vacuum sealing ester used in this test is Apizon H. (4) The dilatometer assembly for weighing the loaded sample. (5) Low-pressure analysis. (6) High-pressure analysis. (7) Cleaning the dilatometer.

### 4.4 Gas Content of the Coal Seam

According to the adsorption isotherm of different coal samples, the methane adsorption by the experimental coal samples (soft coal and hard coal) is consistent with the Langmuir adsorption, which can be expressed as
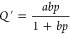
3where *Q*′ is the gas adsorption capacity, cm^3^/g; *p* is the adsorption equilibrium pressure, MPa; and *a* and *b* are adsorption constants, cm^3^/g, MPa^–1^.

At the same time, considering the influence of moisture and ash content in raw coal on gas content, the modified Langmuir adsorption formula is adopted for the adsorbed gas content of raw coal, so the adsorbed gas content of the coal sample at 30 °C and gas pressure *p* can be expressed as follows
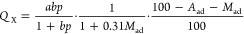
4where *Q*_X_ is the adsorption capacity of raw coal, cm^3^/g.

The free gas exists in the pores of the coal seam. Although the pore volume of coal and the content of free gas are small when the gas pressure is low, the proportion of free gas content in the coal body will increase with gas pressure. Free gas in coal seam follows the gas state equation, which can be expressed as follows
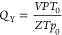
5where *Q*_Y_ is the volume of free gas, cm^3^/g; *V* is the pore volume, cm^3^/g; *T*_0_ is the temperature under standard condition, 273.15 K; *p*_0_ is the pressure under standard condition, 0.101325 MPa; and *Z* is the compression coefficient of methane under gas pressure *p* and temperature *T*, dimensionless.

The actual methane compression factor *Z* is calculated by using the R–K method:
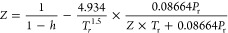
6where *h* is the intermediate variable; *T*_r_ is the gas contrast temperature, *T*_r_ = *T*/*T*_c_; *P*_r_ is the gas contrast pressure, *p*_r_ = *p*/*p*_c_; *T*_c_ is the critical temperature of methane, 190.7 K; and *P*_c_ is the critical pressure of methane, 4.64 MPa.

Coalbed methane generally exists in the adsorbed state and free state, so the gas content of raw coal needs to consider adsorption and free quantity, and then, the gas content of coal under the equilibrium state is calculated by Formula 7.
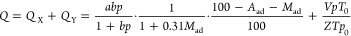
7

**Figure 10:**
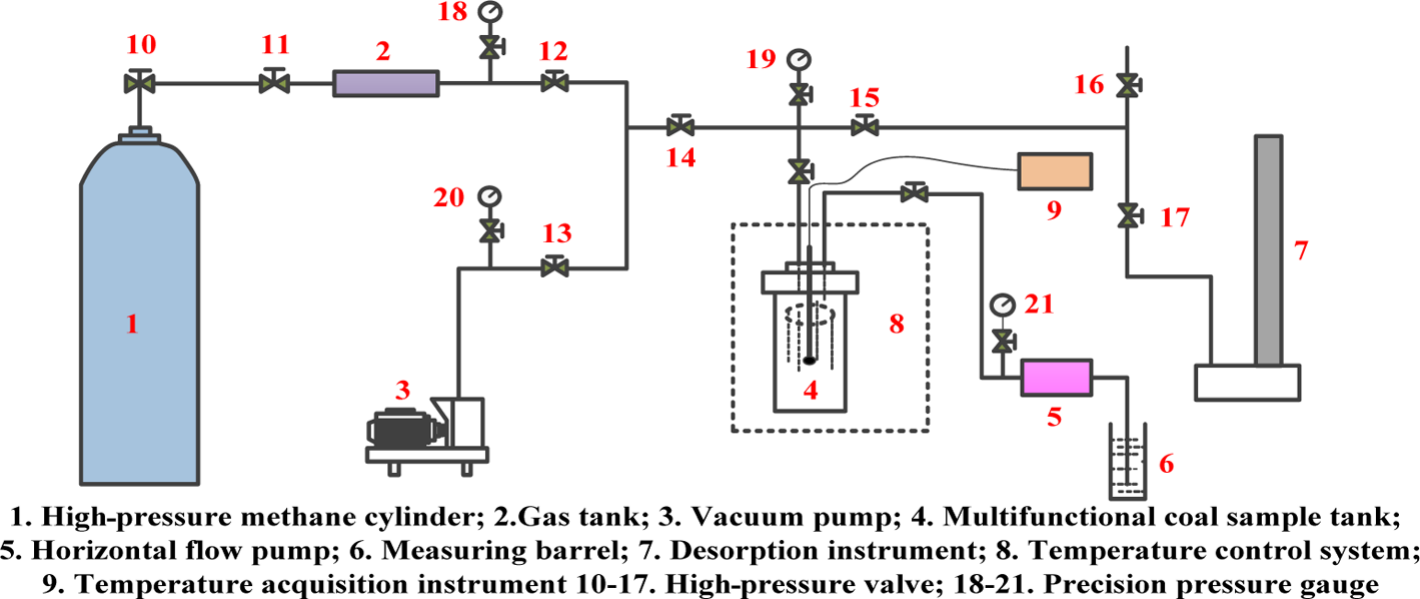
Schematic diagram of the isothermal adsorption device.

The self-made gas adsorption experimental device is composed of the constant temperature adsorption system, vacuum extraction system, and gas quantitative system, as shown in Figure 10. The experimental process is as follows: (1) making a coal sample with a particle size of 60–80 mesh. (2) Setting of the experimental temperature. Temperature control system 8, as shown in Figure 10. The water bath tank is filled with water. (3) Checking the tightness of the experimental device. (4) Measurement of the free space volume. (5) Determination of adsorption capacity. The reference tank is filled with high-purity methane. The balance valve is opened, and isothermal adsorption test is conducted for 24 h. The equilibrium pressure is recorded after the adsorption equilibrium every 60 s, and the test of adsorption capacity is conducted at the pressure point. The isothermal adsorption test is a process of pressurization–equilibrium–pressurization.
